# Laparoscopic biopsy in patients with abdominal lymphadenopathy

**DOI:** 10.4103/0972-9941.30681

**Published:** 2007

**Authors:** D S Bhandarkar, R S Shah, A N Katara, M Shankar, V A Chandiramani, T E Udwadia

**Affiliations:** Department of Minimal Access Surgery, P. D. Hinduja National Hospital, Veer Savarkar Road, Mahim, Mumbai, India; * Department of Pediatric Surgery, P. D. Hinduja National Hospital, Veer Savarkar Road, Mahim, Mumbai, India; ** Department of General Surgery, P. D. Hinduja National Hospital, Veer Savarkar Road, Mahim, Mumbai, India

**Keywords:** Abdomen, biopsy, laparoscopy, lymph node, lymphoma, tuberculosis

## Abstract

**Background:**

Abdominal lymphadenopathy (AL) - a common clinical scenario faced by clinicians - often poses a diagnostic challenge. In the absence of palpable peripheral nodes, tissue has to be obtained from the abdominal nodes by image-guided biopsy or surgery. In this context a laparoscopic biopsy avoids the morbidity of a laparotomy.

**Aim:**

This retrospective analysis of prospectively collected data represents our experience with laparoscopic biopsy of abdominal lymph nodes.

**Materials and Methods:**

Between October 2000 and November 2005, 28 patients with AL underwent laparoscopic biopsy. Pre-operative radiological imaging studies had identified a nodal mass in 20, a solitary node in 1, a cold abscess in 1 and a mesenteric cystic lesion in 1 patient. In five patients with chronic right lower abdominal pain and normal ultra-sonographic findings mesenteric nodes were identified and biopsied during diagnostic laparoscopy.

**Results:**

The sites of biopsied lymph nodes included para-aortic (10), mesenteric (8), external iliac (3), left gastric (2), obturator (1), aorto-caval (1) and porta hepatis (1). One patient with enlarged peripancreatic nodes mass and another with a mesenteric cystic mass had cold abscesses drained in addition to biopsy. There were no perioperative complications and the median postoperative stay was 2 days (range 1-4 days). Histopathology revealed tuberculosis in 23 patients, reactive adenitis in 2, lymphoma in 1 metastatic carcinoma in 1, and a retroperitoneal sarcoma in 1.

**Conclusions:**

In patients with AL, laparoscopy provides a safe and effective means of obtaining biopsy. It is of particular value in patients in whom (a) the nodes are small or present in locations unsuitable for image-guided biopsy, (b) adequate tissue cannot be obtained by image-guided biopsy or (c) previously undiagnosed lymphadenopathy is encountered during diagnostic laparoscopy.

## INTRODUCTION

A patient presenting with abdominal lymphadenopathy (AL) is a common scenario in clinical practice. In those who have associated peripheral lymphadenopathy (PL) a biopsy, which can often be performed under local anesthesia, often yields the diagnosis. However, in the absence of PL diagnostic tissue is more difficult to obtain.

Image-guided needle biopsy (IGNB), whenever feasible, represents a minimally invasive method of obtaining tissue from enlarged abdominal lymph nodes. However, diagnosis may be difficult when needle biopsy yields inadequate tissue or tissue that has lost its architecture. In patients with nodes situated in close proximity to major blood vessels or important viscera, IGNB may be hazardous. Moreover, in patients suspected to have lymphoma, IGNB fails to provide adequate tissue specimen to allow special tests for accurate sub-classification.

Surgeons experienced in laparoscopic surgery are able to biopsy lymph intra-abdominal at almost all locations including those from areas that are considered difficult to access by IGNB. Moreover, laparoscopy affords the surgeon an opportunity to examine the entire abdominal viscera. In this study we review our experience with the use of laparoscopic biopsy in patients with AL.

## MATERIALS AND METHODS

**Figure 1:**
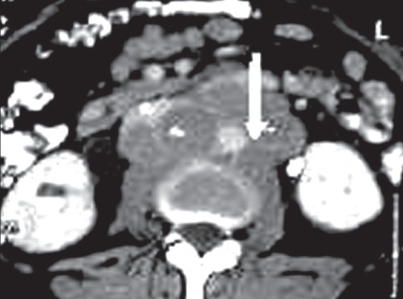
CT scan showing a para-aortic lymph nodal mass (arrow)

Between October 2000 and January 2006, 28 patients underwent laparoscopic biopsy of AL. We retrospectively analyzed prospectively collected data on these patients. There were nine men and 19 women with a median age of 27 years (range 6-77 years). The presenting features included chronic abdominal pain (7), pain, weight loss and fever (10), pain and weight loss (3), abdominal lump (2), pyrexia of unknown origin (5), and backache (1). None of these patients had palpable peripheral lymph nodes suitable for biopsy. In 23 patients, pre-operative ultrasound scan and/or computerized tomography (CT) scan had identified the site of lymphadenopathy [Figure 1]. One patient was shown to have a lesion suspected to be a mesenteric cyst. Ten patients had an earlier IGNB; in nine the tissue obtained was non-diagnostic and in the one patient in whom the biopsy revealed lymphoma, the tissue was considered inadequate for sub-classification. Eleven patients were considered poor candidates for image-guided biopsy as the enlarged lymph nodes were present in unsuitable locations (7) or small (4). In five patients presenting with chronic right lower abdominal pain and having normal imaging studies, mesenteric lymph nodes were identified at diagnostic laparoscopy. In one patient who was empirically started on anti-tubercular therapy upon identification of mesenteric nodal mass, a biopsy became necessary four months later when the response was found to be poor.

### Operative technique

All operations were carried out under general endotracheal anesthesia with the patients placed in modified Lloyd-Davis, left lateral, right lateral or Trendelenburg positions to optimally expose the site of identified lymphadenopathy. A nasogastric tube and Foley catheter were inserted as appropriate; both were removed at the end of surgery.

For upper abdominal procedures a 10-mm camera port was placed slightly above the umbilicus and a 5-mm working port in each mid-clavicular line. In addition, a self-retaining retractor was set up to retract the left lobe of liver. The para-aortic nodes were biopsied by placing the camera port to the right of the midline at the level of umbilicus and two working ports in the midline on either side. For biopsy of the external iliac lymph nodes, the camera port was placed at the umbilicus along with two 5-mm port in para-rectus positions. In patients with chronic abdominal pain undergoing diagnostic laparoscopy a 10-mm camera port was placed at the umbilicus and two 5-mm ports were placed in supra-pubic region and left iliac fossa.

**Figure 2:**
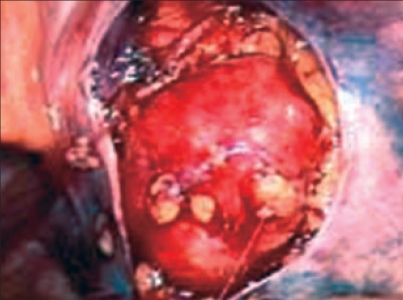
External iliac node being dissected

In all patients a preliminary exploratory laparoscopy was undertaken to examine the peritoneal surface, bowel and liver. The bowel and viscera were displaced from the site of lymphadenopathy by further tilting the table. The peritoneum overlying the lymph nodes was incised and the surface cleared to create a wide window. Either a complete node (when the nodes were discrete) or two to three incisional biopsies (in cases with nodal masses) were obtained [Figure 2]. After the biopsy, hemostasis at the cut edges of the lymph node was secured with electrocautery. Two patients underwent treatment of cold abscesses. In one patient a pre-operatively identified peripancreatic and in another an unsuspected mesenteric abscess was drained, cavities were irrigated and suction drains placed. In all patients the specimens were removed either with the help of a 10-mm cup forceps or after placing them in an impervious plastic bag. The fascia at the site of all 10-mm ports was closed under vision.

## RESULTS

Laparoscopic lymph node biopsy was completed in all patients. Table 1 shows the findings on imaging, sites of lymphadenopathy at laparoscopy, procedures performed, and histopathological diagnosis reached. Laparoscopy had not identified peritoneal or bowel lesions in any of the patients in whom the histology of the nodes subsequently revealed tuberculosis. The median operative time was 45 minutes (range 30-90). There were no intra-operative or post-operative complications and the blood loss was less than 30 mL in all patients. All patients were given parenteral analgesia for the first 24 hours post-operatively and most required minimal oral analgesia thereafter. All patients except the two who had drainage of cold abscesses were discharged after 2 days; the latter two patients stayed in the hospital for 4 days. There were no intra- or post-operative complications.

**Table 1:** Imaging and laparoscopy findings, procedures performed, and final diagnosis

USG/Computerized tomography	Laparoscopic findings	Procedure	Histopathology of nodes
Normal USG (5)	Mesenteric nodes	Lap. appendicectomy + nodal biopsy	TB (3), Reactive (2)
Nodal mass (20)			
Para-aortic (9)	Nodal mass (9)	Biopsy (9)	TB (7)
Mesenteric (3)	Nodal mass	Biopsy + adhesiolysis	Lymphoma (1)
External iliac (3)	Nodal mass	Biopsy	Sarcoma (1)
Left gastric (2)	Nodal mass	Biopsy	TB (3)
Obturator (1)	Nodal mass	Biopsy	TB (3)
Aorto-caval (1)	Nodal mass	Biopsy	TB (2)
Porta hepatis (1)	Nodal mass	Biopsy	Metastatic (1)
			TB (1)
			TB (1)
Peripancreatic mass with abscess (1)	Nodal mass with cold abscess	Drainage of cold abscess with biopsy	TB (1)
Solitary node (1)	Solitary node	Biopsy	TB (1)
Mesenteric cyst (1)	Cold abscess	Drainage of cold abscess with biopsy	TB (1)

Figures in parentheses indicate number of patients. USG - Ultrasonography, TB -Tuberculosis, JJ - Double J

Out of the 23 patients diagnosed to have tubercular lymphadenopathy, 22 were treated with first-line anti-tubercular drug therapy. In one patient undergoing laparoscopic biopsy of a mesenteric nodal mass responding poorly to empirical anti-tubercular therapy, the organisms were found to be susceptible to second-line anti-tubercular drugs. Two patients in whom the mesenteric lymph nodes revealed reactive changes received no further treatment. One patient who had previously undergone surgery for carcinoma of cervix and was found to have metastatic obturator nodes refused further therapy. The patient with lymphoma underwent chemotherapy. This patient at original presentation had a serum creatinine of 1.8 mg% along with left-sided hydronephrosis and hydroureter. A JJ stent inserted at the time of nodal biopsy was removed 3 months later after regression of the nodes following chemotherapy.

## DISCUSSION

The common clinical scenarios in which AL manifests include a palpable mass, pyrexia of unknown origin or lymphadenopathy identified on imaging studies or at laparotomy/laparoscopy. Common causes of AL are metastases, tuberculosis, lymphoma, sarcoidosis, and non-specific inflammation.

In the developing world lymphadenopathy is a common manifestation of abdominal tuberculosis. Although, ultra-sonography and CT are useful in identifying AL, imaging findings may not always disease-specific. Nodes with low density centers, although characteristic of tuberculosis, are not pathognomonic and nodal calcification suggestive of tuberculosis can also be observed in metastases from testicular teratoma and non-Hodgkin's lymphoma.[1] Thus, acquisition of a tissue sample is vital for accurate diagnosis. In skilled hands ultrasonographically guided fine needle aspiration cytology[2] or CT-guided needle biopsy[3] can yield tissue samples adequate for diagnosis. In our series, more than a third of the patients (11/28) were considered poor candidates for IGNB and in further ten patients laparoscopic biopsy was required despite previous IGNB as the tissue obtained was nondiagnostic (9) or more tissue was sought (1). Starting patients diagnosed with AL on empirical anti-tuberculous therapy - as was done in one of our patients - is a practice fraught with the danger of missing out on or delaying the diagnosis of a more sinister pathology. Obtaining a substantial sample is mandatory in patients suspected to have lymph nodal tuberculosis resistant to the first-line anti-tubercular drugs for bacteriological culture and antibiotic sensitivity. Pus in cold abscesses developing in relation to abdominal nodes is often thick and loculated, thus making it unsuitable for imaged-guided drainage. Traditional therapy involves laparotomy and drainage but laparoscopic drainage allows clearing up of all loculi as also confers upon the patient all the benefits of a minimally invasive approach.[4]

Another common indication for laparoscopic biopsy of abdominal lymph nodes reported in the literature is establishment of diagnosis and sub-classification of lymphoma. In experienced hands, use of CT-guided core biopsy of abdominal lymph nodes located in favorable locations can provide adequate samples. However, IGNB techniques may yield inadequate tissue and on occasions, presence of nodes adjacent to major blood vessels or important viscera may render this modality hazardous. Asoglu *et al.* attempted laparoscopic biopsy in 94 patients and completed it successfully in 78.[5] A laparotomy was required in 16 patients (17%) due to inadequate exposure, insufficient tissue, or post-operative adhesions. Lymphoma was diagnosed in 69 patients - in 55 (80%) via laparoscopy, in 9 (13%) via laparotomy and in 5 (7%) with later procedures. Of the remaining 25 patients, 7 had non-lymphomatous disease and 18 had benign lymphadenopathy. The false-negative rate for the laparoscopic procedures was 6%. One patient required conversion to laparotomy for intra-operative hemorrhage. Several other authors have reported successful use of laparoscopy for obtaining biopsy material from abdominal lymph nodes in patients with lymphoproliferative disorders.[6,7] Ability to excise a complete lymph node without having to resort to a laparotomy stands out as the single significant benefit of laparoscopic biopsy in the clinical setting of suspected lymphoma.

In patients undergoing diagnostic laparoscopy for chronic right lower abdominal pain, previously unidentified small mesenteric nodes may be picked up. We always excise completely a representative node in such a situation. Three of five patients who underwent biopsy of incidentally detected mesenteric nodes during diagnostic laparoscopy were found to have lymph nodal tuberculosis. As all these patients had longstanding right lower abdominal pain, an appendicectomy was performed concurrently to avoid future diagnostic confusion.

Lymph nodes identified on imaging studies in patients being investigated for pyrexia of unknown origin (PUO) forms yet another indication for laparoscopic lymph node biopsy. Arch-Ferrer *et al.* reported 15 patients with PUO who underwent diagnostic laparoscopy.[8] Tissue samples were obtained from liver, spleen and lymph nodes which allowed an etiologic diagnosis to be reached in 10 patients and in ruled out abdominal pathology as cause for the PUO in four others. Thus, 93% of the patients undergoing laparoscopy were benefited by the procedure.

Some of the technical aspects of laparoscopic biopsy of abdominal lymph nodes merit elucidation. A careful study of the CT scan helps in planning the approach to the target area and position of ports. Proper positioning of the patient allows the bowel and other viscera to fall away from the site of lymphadenopathy. A liver retractor may have to be placed when working in the upper abdomen. Large nodal masses are readily apparent through the overlying peritoneum but in obese patients discrete nodes may sometimes be hidden in fatty tissue making their identification difficult. A generous incision on the peritoneum and a careful search usually reveals the nodes even when solitary or small. Whenever feasible, we prefer to obtain at least one complete node, but in presence of a nodal mass a block of tissue no less than 1 × 1 cm is excised. During excision/biopsy of the node use of electrocautery is kept to a minimum as its excessive use damages the nodal architecture making the histopathological interpretation difficult. Swabbing with a small piece of gauze introduced in the peritoneal cavity is used for maintaining a blood-free operative field. We prefer to extract the biopsy specimen with the use of a 10-mm cup forceps or by placing it inside a plastic bag so as not to crush/damage it. Whenever available, intra-operative frozen section is obtained to confirm adequacy of the specimen.

Our series does not represent all patients with AL seen at the institution during the study period. Majority were patients in whom an IGNB had failed or was not feasible thus leading to a referral for laparoscopic lymph node biopsy. It is likely that in several other patients either biopsy of palpable peripheral nodes or an IGNB had provided the diagnosis. Thus analysis of our data is unable to provide an insight into the percentage of patients with AL who may go on to require a laparoscopic biopsy.

## CONCLUSION

To summarize, laparoscopic biopsy represents a minimally invasive method of obtaining tissue from enlarged abdominal nodes present in locations unsuitable for IGNB, when tissue obtained by IGNB is inadequate and when previously unsuspected lymphadenopathy is identified during diagnostic laparoscopy. This method allows a surgeon experienced in laparoscopic technique to obtain adequate-sized biopsy specimen under visual control from lymph nodes in almost any intra-abdominal location. With its easy availability, early and judicious use of laparoscopic biopsy should be considered in the algorithm of work up of patients with AL.
